# Molecular characterization of imprinting disorders: Beckwith–Wiedemann, Silver–Russell, and Prader-Willi syndromes in Egyptian patients

**DOI:** 10.1186/s12887-025-05901-4

**Published:** 2025-07-29

**Authors:** Amal M. Mohamed, Ola Eid, Marwa Farid, Engy Ashaat, Ghada M. H. Abdel-Salam, Hala T. El-Bassyouni, Mahmoud Essa, Rana Mahrous, Peter S. F. Erian, Khaled M. Refaat, Alaaeldin Fayez, Maha Zaki

**Affiliations:** 1 Institute of Human Genetics and Genome Research, Department of Human Cytogenetics, National Research Centre, Cairo, Egypt; 2 Clinical Genetics Department, National Research Centre, Cairo, Egypt; 3 Molecular Genetics and Enzymology Department, National Research Centre, Cairo, Egypt

**Keywords:** Imprinted disorders, Beckwith Wideman, Silver-Russel syndrome, Prader-Willi, Single nucleotide polymorphism analysis, Imprinted genes, Hypo and hyper methylation, Uniparental disomy

## Abstract

**Background:**

Imprinted genes, characterized by monoallelic expressions (either maternal or paternal), they are crucial for normal growth and development. Disruption of their monoallelic expressions leads to imprinting disorders (ImpDis). The aim of this study is to achieve proper diagnosis of ImpDis in Egyptian patients through clinical evaluation and genetic testing, emphasizing certain clinical manifestations that may indicate ImpDis to provide accurate diagnosis and genetic counseling.

**Methods:**

Fifty-three patients, either clinically evaluated for Impaired Disposition (ImpDis) or suspected to have it, were referred from the outpatient genetic clinics at the National Research Center, Egypt. Nineteen patients displayed clinical manifestations of ImpDis syndromes, while 34 showed signs affecting growth, which suggested ImpDis. These growth-related symptoms included growth retardation, feeding problems, failure to thrive, hypoglycemia, obesity, hemihypertrophy, asymmetry, and overgrowth. Of the 19 patients with syndromic ImpDis, 8 were clinically diagnosed with Silver-Russell syndrome (SRS), 7 with Prader-Willi syndrome (PWS), and 4 with Beckwith-Wiedemann syndrome (BWS). We employed methylation-specific multiple ligation-dependent probe amplification (MS-MLPA) for all patients, SNP-array testing for 12 patients, and whole exome sequencing (WES) for one patient.

**Results:**

In patients with Silver-Russell syndrome (SRS), one patient exhibited hypermethylation of the *GRB10* and *MEST* genes, along with segmental uniparental disomy (UPD) on chromosome 7 (patient 1). Another patient had a variant in the *HMGA2* gene (NM_001300918.1:c.310dup), which, according to the American College of Medical Genetics (ACMG) criteria, was classified as PM2 VUS (patient 2). In patients with Prader-Willi syndrome (PWS), one patient showed hypermethylation of the *SNPRN* gene (patient 3). In patients with Beckwith-Wiedemann syndrome (BWS), two displayed hypomethylation of the *KCNQ-CR* region (patients 4 and 5). Among the group of patients with symptoms suggestive of ImpDis, no methylation defects were detected through MS-MLPA.

**Conclusion:**

It is crucial to diagnose ImpDis accurately, as understanding the exact cause of ImpDis is important for genetic counseling and personalized medicine. Early diagnosis enables timely interventions, which can improve developmental outcomes. Precision in diagnosis helps differentiate between conditions with overlapping clinical features. *HMGA2* mutation should be verified in SRs patients with negative 11p15 methylation defect and matUPD7.

## Background

Imprinted genes are characterized by monoallelic expression, meaning they are expressed from either the maternal or paternal allele, but not both. These genes are crucial for normal development, and disruptions in their monoallelic expression can lead to imprinting disorders (ImpDis). Over 100 imprinted genes have been identified in humans and mice [1, 2].

Imprinting control regions (ICRs) are specific DNA sequences that regulate the imprinting of nearby genes. These regions determine whether a gene will be expressed from the maternal or paternal allele. ICRs are typically located near imprinted genes and play a key role in controlling DNA methylation and histone modifications.

Differentially methylated regions (DMRs) are regions in the genome where DNA methylation marks differ between the maternal and paternal alleles. DMRs can be located within the imprinted genes themselves or within regulatory regions such as ICRs [3]. While ICRs directly regulate imprinting by controlling DNA methylation patterns, DMRs primarily reflect differences in DNA methylation at specific loci, which can lead to the silencing or activation of imprinted genes.

Imprinted genes play critical roles in embryonic growth, placental formation, and metabolism. Aberrant expression of these genes can result from epigenetic or genetic abnormalities, including aberrant hypomethylation or hypermethylation at ICRs, uniparental disomies, chromosomal aberrations (deletions, duplications, translocations, inversions of imprinting domains), and point mutations of imprinted genes [4].

In Silver-Russell Syndrome (SRS) Hypomethylation of the H19/IGF2 imprinted domain on chromosome 11p15 accounts for 50–60% of cases of Silver-Russell Syndrome (SRS), while maternal uniparental disomy of chromosome 7 (MatUPD7) is found in about 10% of cases. The genetic cause remains unknown in 30% of clinical SRS cases. Other reported genetic causes include uniparental disomy of chromosome 20 and mutations in imprinted genes such as I*GF2*, *CDKN1C*, and *PLAG1* [5]. Additionally, recent findings have identified mutations in the non-imprinted *HMGA2* gene as a monogenic cause of SRS [6–9].

PWS is caused by the lack of expression of paternal genes on chromosome 15q11.2-q13.1. In 70% of patients, PWS is due to a deletion on the paternal chromosome 15q11.2-q13.1. In 20–30% of cases, maternal UPD of chromosome 15 causes PWS. In about 3% of cases, the cause is a deletion of the imprinting center [10].

In Beckwith-Wiedemann Syndrome (BWS) the loss of methylation (LOM) at the *KCNQ1OT1* on chromosome 11p15.5 (within imprinting center 2 [IC2]) is maternally imprinted and represents the cause in 50–60% of patients. This leads to the biallelic expression of *KCNQ1OT1* [11]. The presence of uniparental disomy (UPD) of 11p15.5 accounts for 20–25% of BWS cases. Additionally, gain of methylation (GOM) in IC1 is found in less than 4% of cases [12]. Point mutations in the imprinting center are rare, occurring in 1.3–5% of cases. Duplication of the paternally inherited 11p15.5 can cause BWS in less than 1% of cases [13–15].

The proper diagnosis of imprinting disorders (ImpDis) is challenging due to overlapping phenotypes and molecular heterogeneity [16]. The clinical and molecular overlap between ImpDis suggests causal links between them, either through shared causes of dysregulation affecting multiple imprinted genes or by perturbation of interactions between the products of imprinted genes [17–19].

A subgroup of patients with imprinting disorders exhibits methylation changes at more than one imprinted locus, a phenomenon known as multi-locus imprinting disturbance (MLID). This condition is observed in some patients with classic manifestations of imprinting disorders (ImpDis), while others present with clinical features that overlap multiple imprinting disorders. In some cases, MLID is found in patients with atypical clinical features of ImpDis [20–22]. The processes of MLID are unknown. The diagnosis of MLID has important implications for both management and prognosis [22].

Another hypothesis explaining the clinical and molecular overlap between different ImpDis is the"imprinted gene network"(IGN) [23]. Recently, additional gene networks have been described, including the role of unoccupied insulin receptors (IR) and insulin-like growth factor 1 receptor (IGF1R) signaling in the coordinated regulation of multiple imprinted genes associated with growth and development in mice [24].

## Methods

We aimed for proper diagnosis of ImpDis in Egyptian patients, depending on clinical evaluation and genetic testing, and emphasized certain clinical manifestations that may indicate ImpDis to provide accurate diagnosis and genetic counseling.

The study included 53 patients, divided into two groups. The first group comprised 19 patients diagnosed with syndromic imprinting disorders (ImpDis), all of whom exhibited the characteristic clinical manifestations of ImpDis. This group included 8 patients with Silver-Russell syndrome (SRS), 7 patients with Prader-Willi syndrome (PWs), and 4 patients with Beckwith-Wiedemann syndrome (BWs). The second group displayed signs of growth disturbances suggesting imprinting disorders, such as growth retardation, feeding problems, failure to thrive, hypoglycemia, obesity, hemihypertrophy, asymmetry, and overgrowth.

The study was approved by the ethical committee of the National Research Centre. Certificate number: 19250.

The research Funded by the National Research Centre: Project No.: 12060189.

We followed the criteria for diagnosis in different international consensus for Beckwith-Wiedemann, Silver-Russell, and Prader-Willi syndromes [12, 25, 26] to reach for accurate diagnosis and provide accurate genetic counseling.

- DNA extraction using BAX gene tubes and kits.
- Karyotype analysis was performed to all patients to detect any balanced translocations that may affect the imprinting centers, In patients with PWs we performed karyotype for the parents.
- Methylation specific multiple ligation probe amplification (MS-MLPA): We applied multiple ligation probe amplification (MS-MLPA) to all patients using MS-MLPA Probemix ME034-B1 Multi-locus Imprinting. The method was performed according to the manufacturer’s instructions. (ME034-B1 Multi-locus Imprinting-MRS-Holand) [27]. This kit is designed to detect abnormal methylation and copy number variations in one or more sequences within imprinted regions. Following the manufacturer's instructions, we denatured the DNA and performed an overnight hybridization with the MLPA probemix. This was followed by digestion, probe ligation, and amplification. The amplified products were separated using an ABI 3500 Genetic Analyzer (USA), and data analysis was conducted using Coffalyser.Net software (MRC-Holland).
- SNP array was performed for some selected patients (12 patients), especially those with imprinting defects to detect if the cause is due to uniparental disomy or other CNVs that may have influenced the imprinting center. We used Cytoscan HD Microchip (Affymetrix, Santa Clara, CA, USA, Thermo Fisher Scientific, Inc.;Waltham, MA, USA) [28, 29].

According to the manufacturer’s instructions, our workflow started with DNA digestion, ligation and PCR amplification. The PCR product purified with magnetic beads, fragmented, labeled with biotin, injected to the cytoscan HD microchips and incubated at 60◦C for 16 h in hybridization oven (Thermo Fisher). After incubation, the microchips were washed and stained with streptavidin using the GeneChip Fluidics Station 450 (Thermo Fisher Scientific). The microchips scanned by the GeneChip Scanner 3000 (Thermo Fisher). We used command console software (ChAS) for data analysis.

### WES was performed for patient 2

DNA Library was generated after Genomic DNA is enzymatically fragmented, and target regions are enriched using DNA capture probes. The library contained more than 98% of the coding RefSeq according to the human genome build GRCh37/hg19 (about 41 Mbs of > the human coding exome including the mitochondrial genome). DNA samples were sequenced on an Illumina platform to obtain at least 20 × coverage depth for > 98% of the targeted bases according to the manufacturer’s protocol. The quality of the generated FASTQ files was analyzed with FastQC software (version 0.11.5; Illumina, San Diego, CA, USA). Variant Annotation and Filtration PhenoDB tool were used to annotate VCF files using ANNOVAR [30]. Variants were filtered based on the depth of coverage and minor allele frequencies less than 1% MAF in dbSNP [31], 1000 Genomes Project [32], and the Genome Aggregation Database (gnomAD v2.1.1). The investigation for relevant variants is focused on coding exons and flanking ± 10 intronic nucleotides of genes with clear gene-phenotype evidence in Human Genome Mutation Database (HGMD) and ClinVar. The provided family history and clinical information are used to evaluate identified variants along all the potential inheritance modes. According to the ACMG guidelines, all variants are categorized into five classes (pathogenic, likely pathogenic, variant of uncertain significance (VUS), likely benign, and benign). Mitochondrial variants are reported for heteroplasmy levels of 15% or higher. The copy number variation (CNV) with sensitivity of more than 95% for all homozygous/hemizygous and heterozygous deletions/duplications spanning at least three consecutive exons was considered. Variants naming was checked using Mutalyzer v 3.0.8 [33]. Determination the effect of studied variants was carried using Ensembl Variant Effect Predictor (VEP) server [34].

#### Clinical evaluation of imprinting disorders

The study included 53 patients, divided into two groups. The first group comprised 19 patients diagnosed with syndromic imprinting disorders (ImpDis), all of whom exhibited the characteristic clinical manifestations of ImpDis.

Eight patients exhibited clinical manifestations of Silver-Russell syndrome (SRS). We followed the consensus guidelines for SRS diagnosis [9, 35] and adhered to the clinical criteria for SRS. Patients were clinically diagnosed with SRS if they met four of the six criteria in the Nechine-Harbison classification [36]: prenatal growth retardation, postnatal growth retardation, relative macrocephaly at birth, protruding forehead, body asymmetry (e.g., leg length discrepancy or arm asymmetry, with at least two other asymmetries not including the face), feeding difficulties, and low body mass index (BMI) at two years. When a patient fulfilled four of the NH-CSS criteria, SRS was suspected, and genetic testing was warranted. All our SRS patients met the clinical criteria for SRS.

Seven patients exhibited clinical manifestations of Prader-Willi syndrome (PWS). We followed the consensus diagnostic criteria and recommendations [37, 38]. The diagnosis of PWS depends on specific clinical criteria that vary with age. These criteria include major, minor, and supportive factors [33]. For patients under three years old, a score of five (including four major criteria) is required for diagnosis. For patients aged three years to adulthood, a score of eight (including at least five major criteria) is needed.

Major criteria (valued at one point each) include: neonatal hypotonia, feeding problems, weight gain, characteristic facial features with almond-shaped eyes, hypogonadism, global developmental delay, hyperphagia, deletion of chromosome 15q11.2-q13.1.

Minor criteria (valued at half a point each) include decreased fetal movement, behavior problems, sleep disturbance, short stature, skin hypopigmentation, small hands and feet, speech defects, eye anomalies, thick saliva, skin picking.

Supportive criteria (which have no score) include high pain threshold, vomiting, temperature instability, scoliosis or kyphosis, early adrenarche, osteoporosis, unusual skin, normal neuromuscular examination. All our patients that were clinically diagnosed with PWS met the criteria of PWS.

Four patients exhibited manifestations of Beckwith-Wiedemann syndrome. We followed the consensus guidelines for the clinical and molecular diagnosis of BWS [12]. According to the BWS consensus reporting the diagnosis is based on cardinal features (each worth two points) and suggestive features (each worth one point).

Cardinal features (two points each): Macroglossia, exophthalmos, prolonged hyperinsulinism, unilateral overgrowth, Wilms tumor.

Suggestive features (one point each): birth weight above the mean, umbilical hernia, polyhydramnios, hepatomegaly, tumors, ear creases, transient hypoglycemia.

Patients with more than 4 points are diagnosed as BWS. Our patients fulfilled the clinical criteria of BWS.

Thirty-four patients exhibited clinical manifestations affecting growth, suggesting the presence of an imprinting disorder. This included growth retardation, feeding problems, failure to thrive, hypoglycemia, obesity, hemihypertrophy, asymmetry, and overgrowth.

ChatGPT: was used for languishing editing.

## Results

**Fig. 1:**
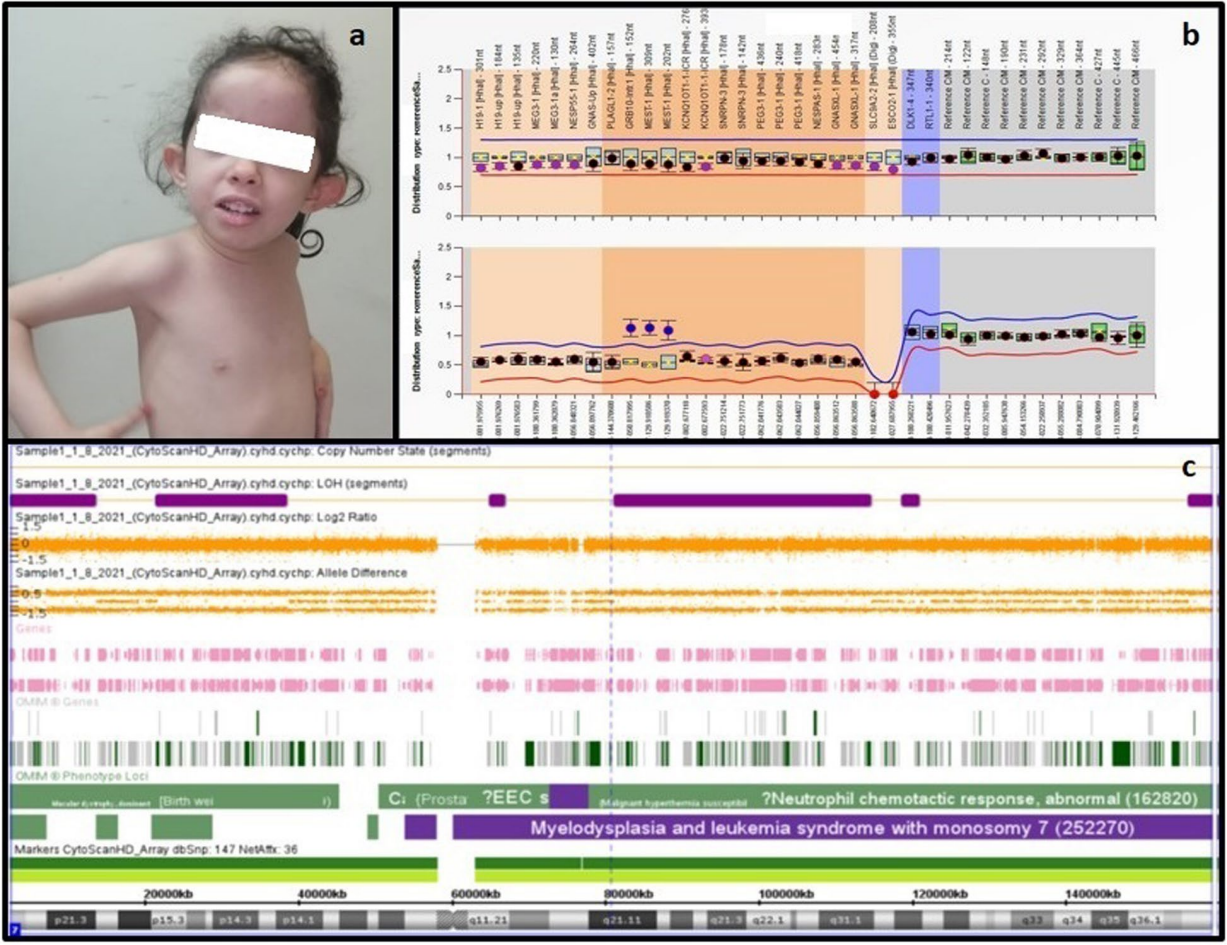
Patient 1: **a** Face with frontal bossing, dolichocephaly- Synophrys—deep seated eye- low set and cupped ears. **b** Ms-MLPA showing normal copy number variant and hypermethylation of *GRB10* and *MEST-1* gene on chromosome. SNP array shows segmental uniparental disomy of chromosome 7

**Fig. 2:**
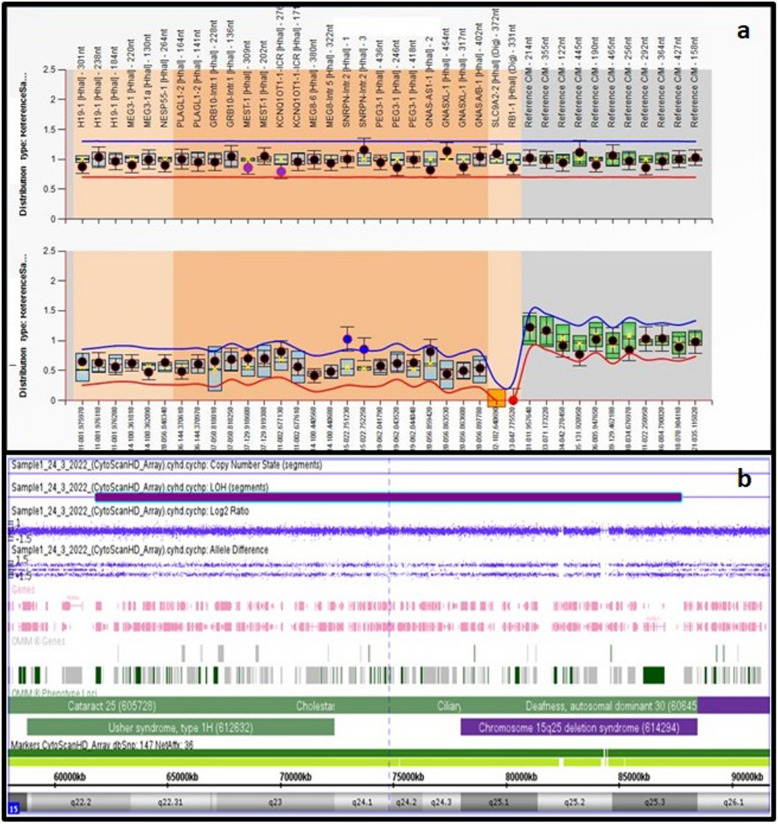
patient 3: **a** MS-MLPA showing hyper methylation of *SNRPN* gene on chromosome 15q11.2 which is maternally imprinted, **b** SNP array showing segmental UPD of chromosome 15

**Fig. 3:**
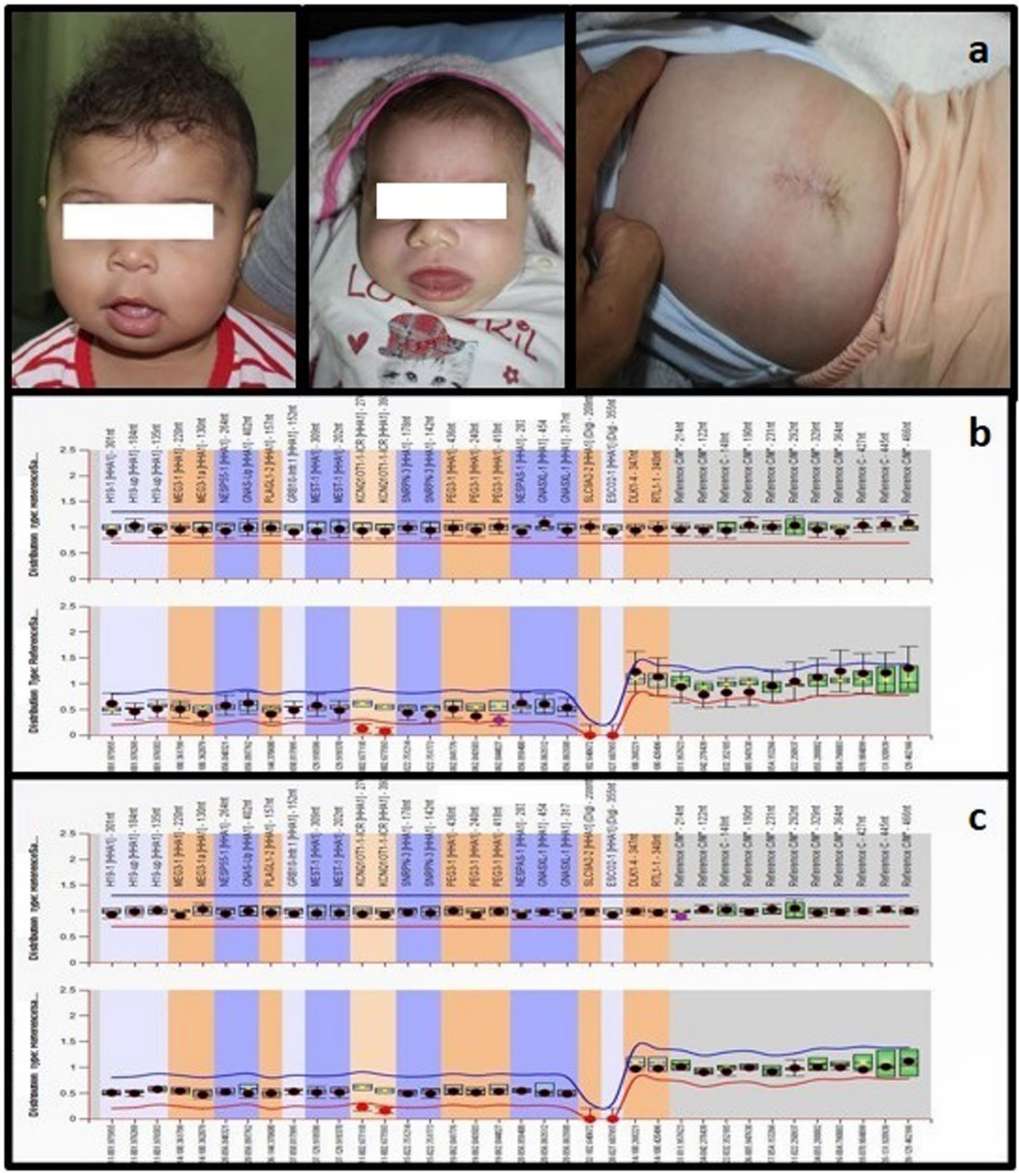
patients 4 and 5: **a** face of patients 5, and 6 with protruded tongue, **b** MS-MLPA showing hypomethylation of *KCNQ1OT1* gene at 11p15.5 which is maternally imprinted

**Fig. 4:**
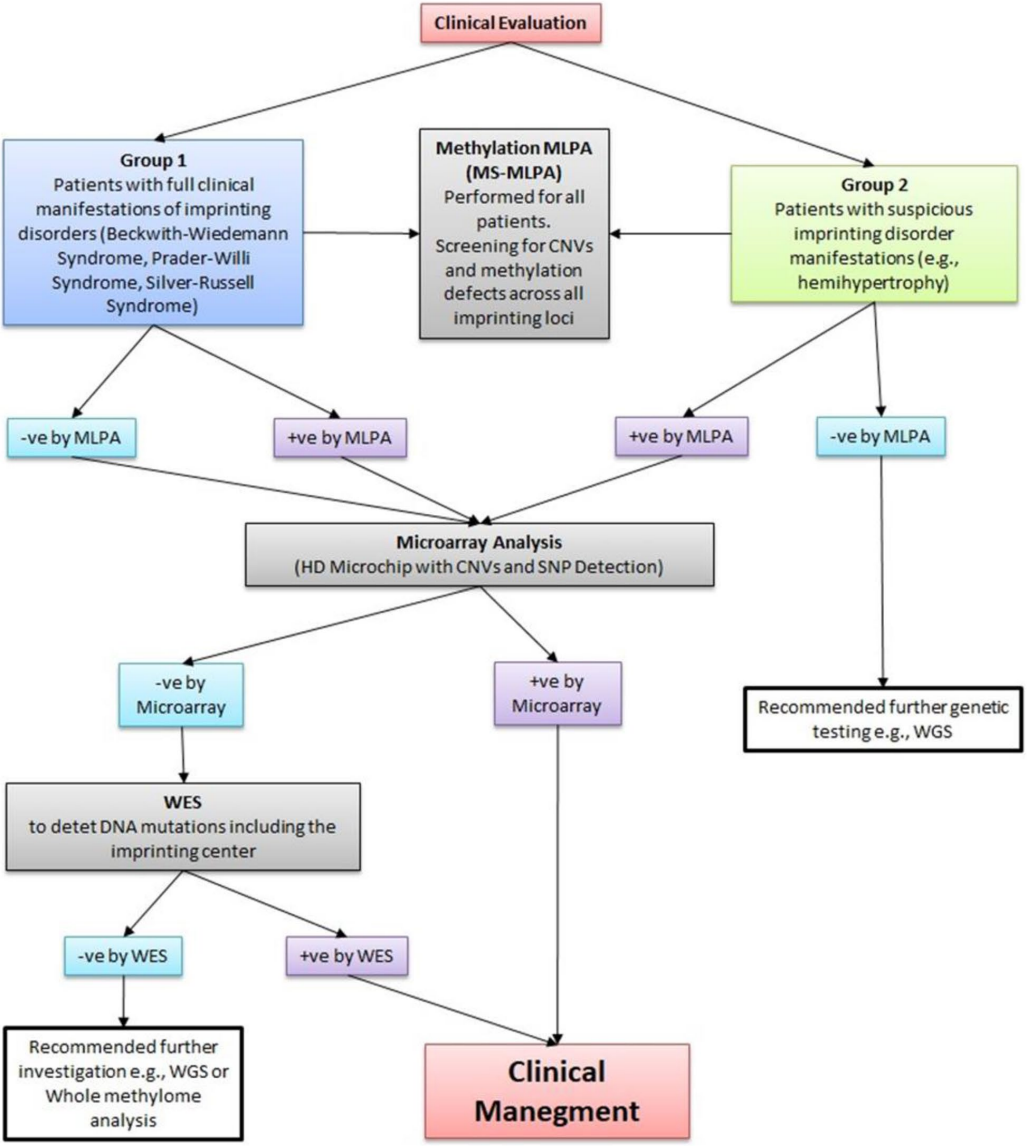
shows the flow chart of investigations in the two studied groups

In Group 1, the clinical manifestations of patients with positive epigenetic and genetic causes are summarized in Table 1. Figures 1, 2 and 3 show the MS-MLPA and SNP array results in patients 1,3,4 and 5. Figure 4 illustrates the flow chart for investigations of ImpDis in our studied cohort.

**Table 1:** Clinical manifestations and diagnostic criteria score in 5 patients with epigenetic and genetic causes for imprinting disorders

	**Patient 1**	**Patient 2**	**Patient 3**	**Patient 4**	**Patient 5**
**Age/Gender**	6y/F	8y/F	9y/F	6 m/F	2 m/F
Consanguinity	-ve	-ve	+	-ve	-ve
low birth weight	+ ve	+ ve	-ve	-ve	-ve
Intrauterine growth restriction	+ ve	+ ve	-ve	-ve	-ve
Feeding problems	+ ve	+ ve	Hyperphagia	-ve	-ve
Delayed milestones	+ ve	+ ve	+ ve	+ ve	+ ve
Growth retardation	+ ve	+ ve	-ve	-ve	-ve
Dysmorphic Features	frontal bossing, dolichocephaly, synophrys, deep-set eyes, low-set cupped ears, clinodactyly	Triangular face, frontal bossing thick eyebrows, short nose, low-set small ears, micrognathia, cleft palate, downturned corners of the mouth	Almond shaped eyes, Fusiform fingers, Truncal obesity, Hypotonia	Epicanthic folds, depressed nasal bridge, Low set ears, long flat philtrum, Macroglossia	Proptosis, depressed nasal bridge, macroglossia
other Clinical Features	Umbilical hernia, Absent labia minora, hypotonia, MRI: dilated ventricles	lower limb asymmetry, areas of hyperpigmentation, learning disability, stuttering	hypoplastic labia minora, Delayed Pubertal staging: Tanner stage B2 A1 P1., Delayed expressive language	Asymmetry in the lower limbs (the left lower limb was 0.5 cm longer than the right), Two café-au-lait patches on the hypertrophied side, Hypotonia	operated congenital exophthalmos, hepatomegaly, Sandal gap between 1 st and second toes, Echocardiography: atrial septal defect (ASD), ventricular septal defect (VSD), and mild pulmonary stenosis, ultrasound revealed mild hepatomegaly
Anthropometric Measurements	Weight: 10 kg,(−3.9 SD), head circumference: 47 cm (−3.2 SD) microcephaly, height: 89 cm (−4.9 SD) short	Under weight, Microcephaly, short stature	Weight (+ 2.52 SD), head circumference: 52 cm (+ 0.15 SD), height: 130 cm (−0.48 SD)	Weight: 7 kg (−0.5 SD), head circumference: 41 cm (−1 SD), height: 66 cm (+ 0.35 SD)	weight: 5.5 kg (+ 1.1 SD), head circumference: 36.5 cm (−0.6 SD), length: 60 cm (+ 1.9 SD)
intelligence quotient (IQ)	67	N/A	65		
Diagnosis	Silver-Russell Syndrome NH-NH-CSS criteria 4/6	Silver-Russell Syndrome NH-CSS criteria 5/6	Prader-Willi syndrome Consensus diagnostic criteria scored 7 of the major criteria, And 2 of minor criteria 8/8	Beckwith-Wiedemann syndrome Consensus diagnostic criteria scored 4	Beckwith-Wiedemann syndrome Consensus diagnostic criteria scored 4

M: month, Y: Year, F: female, -ve: negative, + ve: positive, N/A: not available, NH-CSS: Netchine-Harbison Clinical Scoring System, MRI: magnetic resonance imaging

### Epigenetic and genetic findings

We used the MS-MLPA Probemix ME034-B1 Multi-locus Imprinting for all patients. The 34 patients who exhibited clinical manifestations affecting growth and suggested of imprinting disorder revealed no copy number variance or methylation defect in the imprinting loci. In the group of syndromes of ImpDis, five patients showed positive epigenetic and genetic results, Table 2 provides a summary of the genetic causes associated with syndromic imprinted disorders.

**Table 2:** represents patients with epigenetic and genetic causes for imprinting disorders

Patient No	Clinical presentation	Genetic and epigenetic cause	Chromosome position	Imprinted allele	Array-CGH
1	SRs	hypermethylation of *GRB10*	7p12.1	Maternal	Segmental UPD of chromosome 7
		hypermethylation of *MEST*	7q23.1		
2	SRs	frame shift mutation in *HMGA2* gene	12q14.3	-	Normal
3	PW	hypermethylation of *SNRPN*	15q11.2	Maternal	Segmental UPD of chromosome 15
4	BWs	hypomethylation of *KCNQ-CR*	11p15.5	Maternal	Normal
5	BWs	hypomethylation of *KCNQ-CR*	11p15.5	Maternal	Normal

#### Patient 1

Diagnosed with Silver-Russell syndrome (SRS) using MS-MLPA, which revealed a normal copy number variant and hypermethylation of both *GRB10* (7p12.1) and *MEST (7q23.1) genes.*

Both genes are maternally imprinted region hg18 chr7:g.50,817,995|gom, and hg18 chr7:g.129.918586_ 129.919370|gom (gom: gain of methylation); [39]. SNP array showed segmental uniparental disomy of chromosome 7 (Fig. 1).

#### Patient 2

MS-MLPA showed normal CNV and normal methylation pattern. Whole-exome sequencing (WES) identified a variant in the *HMGA2* gene (NM_001300918.1:c.310dup), p.(His104Profs*12), heterozygous, causing a frameshift of uncertain significance (class 3). WES was performed for her parents and approved that the mutation is de novo.

According to American Collage of Medical Genetics (ACMG) criteria [40] the NM_001300918.1:c.310dup variant was classified as PM2 VUS.

Using Ensemble Variant Effect Predictor (VEP) and Applying nonsense-mediated decay (NMD) assessment to NM_001300918.1:c.310dup variant, it is predicted that the variant is NMD escaping variant (PTVescs).

#### Patient 3

Diagnosed with Prader-Willi syndrome (PWS). MS-MLPA showed hypermethylation of the *SNRPN* gene on chromosome 15q11.2 hg18 chr15:g.22,751,214_22,751,773|gom [39] which is maternally imprinted. SNP array reviled large segment of UPD on chromosome 15 (Fig. 2).

### Patients 4 and patient 5

Both presented with clinical manifestations of Beckwith-Wiedemann syndrome (BWS). MS-MLPA revealed normal copy number variance at chromosome 11p15.5 in both patients, with hypomethylation of *KCNQ1OT1* (KCNQ-CR) at 11p15.5, which is maternally imprinted hg18 chr11:g.2,677,118_2,677.593|lom (lom: loss of methylation). The hypomethylation of *KCNQ1OT1* causes biallelic expression of this gene, leading to BWS (Fig. 3) SNParray show normal female karyotype in patients 4 and 5 arr(X,1–22) × 2.

## Discussion

Imprinting disorders (ImpDis) encompass a broad range of clinical manifestations that often overlap with other conditions. Some patients exhibit mild phenotypes, with modifications of the phenotype occurring as they age. Despite sharing common features, such as growth disturbances and developmental delay, imprinting disorders are highly heterogeneous, and their clinical manifestations are often nonspecific, which complicates the diagnostic process [41–45]. The 1 st group of patients were 8 with SRS, 7 with PWS, and 4 with BWS, they fulfil the clinical scoring of the imprinted disorders ([9, 12, 37]. The second group of patients had symptoms that affect growth. which suggest imprinting disorders, they exhibited feeding problems, failure to thrive, hypoglycemia, growth retardation, obesity, body asymmetry, hemihypertrophy, and overgrowth. In the 2nd group we utilized MS-MLPA to confirm or exclude the presence of imprinting disorders, and while MS-MLPA results were negative, these patients may still harbor imprinting defects in regions outside known imprinting loci or mutations in imprinting centers. Further molecular testing is needed for this group to fully explore potential genetic causes.

Based on cost-effectiveness, we performed cytogenetic analysis and MS-MLPA for all our patients. HD-microarray was used to detect UPD in some selected patients (12patients), and WES was applied for one patient. The study highlights the importance of diagnosing imprinting disorders (ImpDis) through a combination of clinical evaluation and advanced genetic testing. We adhere to the diagnostic consensus for Silver-Russell, Prader-Willi and Beckwith-Wiedemann syndromes to ensure accurate diagnosis, appropriate genetic counseling, and management [9, 12, 37].

Methylation Specific MLPA (MS-MLPA) is an effective tool for detecting methylation alterations and copy number changes (deletions or duplications) at imprinting loci, making it highly useful for diagnosing imprinting disorders in 70% to 90% of cases (27). MS-MLPA is highly sensitive, capable of detecting small changes in methylation status at multiple loci in a single assay, providing a robust screening method for these disorders. Additionally, Methylation Specific MLPA is relatively low-cost compared to other techniques like Whole Exome Sequencing (WES) and is faster than most other genetic testing methods.

However, MS-MLPA is limited to detecting only imprinting defects associated with the known imprinting disorders, it cannot identify unknown imprinting loci, and it does not provide detailed information on the underlying genetic mutations (27).

In contrast, Whole Exome Sequencing (WES) sequences all exonic regions of the genome, detecting variants in the coding regions. While WES is unable to identify methylation changes, it can uncover genetic mutations that may lead to imprinting disorders and other genetic conditions that Methylation Specific MLPA cannot detect [46]. WES can potentially identify unknown mutations contributing to imprinting disorders, but it is more costly and time-consuming than MLPA. Since WES focuses on genetic sequences, it cannot detect DNA methylation alterations and may not be the most efficient approach for diagnosing imprinting disorders, where methylation is the primary cause. In these cases, methylation-based tests like Methylation Specific MLPA or methylation-sensitive PCR are often more direct and cost-effective. In the 1 st group MS-MLPA could detect methylation defects in 4 patients, WES performed for patient 2 and detected a variant in *HMGA2* gene which was classified as PM2 VUS.

To our knowledge this is the 1 st time in Egypt to use MS-MLPA and array CGH to explore the causes of ImpDis.

We applied SNP-array to detect UPD (uniparental disomy) as well as copy number variants (CNVs) that may affect imprinting loci, and to identify low-level mosaicism affecting UPD or CNVs at imprinting gene sites. Additionally, we performed karyotype analysis to diagnose balanced translocations involving imprinting centers.

The application of methylation-specific multiple ligation-dependent probe amplification (MS-MLPA), SNP array, and whole exome sequencing (WES) enabled a more thorough investigation into the genetic causes of imprinting disorders. In our patients with Silver-Russell syndrome (SRS) group, hypermethylation of *GRB10* and *MEST* genes associated with segmental UPD in chromosome 7 detected in patient 1, frameshift mutation in the *HMGA2* gene in patient 2. These findings underscore the complexity of imprinting disorders, where diverse genetic and epigenetic mechanisms result in similar clinical phenotypes. It is reported that epigenetic effect accounts for 70% of the causes of SRS. Mutation in other imprinting genes as as *IGF2, CDKN1C*, and *PLAG1* can cause around 30% *SRS* [5]. Mutation in *HMGA2* gene can cause SRS and this gene must be checked in SRS patients who are found to be negative for 11p15 methylation defect or matUPD7 [7, 47]. Pathogenic variants in the *HMGA2* gene were reported in five patients with Silver-Russell Syndrome (SRS) (8), a variant of uncertain significance in HMGA2 gene described in a boy who had clinical characteristics of SRS (7). In Silico analysis for the variant in our patient 2 demonstrated the presence of a PTVescs variant in the *HMGA2* gene. The NM_001300918.1:c.310dup variant was detected in the fifth and last exon near the 3'untranslated region (3'UTR). Premature termination codons (PTCs) variants near the 3'end of the final coding exon are expected to escape NMD [48]. These variants are called PTVescs and they escape nonsense-mediated decay, thus the mRNA persists in the cell for a longer period than usual without being degraded, causing loss of function impact [49]. Therefore, NM_001300918.1:c.310dup appears to lead to intolerant mRNA of the *HNGA2* gene that is likely to be associated with Russell-Silver syndrome.

In Prader-Willi syndrome (PWS), patient 3 exhibited hypermethylation of SNRPN gene, array CGH detect a large segment of UPD15. In the Beckwith-Wiedemann syndrome (BWS) group, patients 4 and patient 5 displayed hypomethylation in the *KCNQ1* control region, which is observed in approximately 60% of BWS cases.

### Genetic counseling

LOM of chromosome 11p15 represents 30–60% of causes of SRS, this was not detected in our SRS patients, UPD of chromosome 7 represents 5–10% of SRS, it was detected in our patient 1. Parents of a child with maternal UPD chromosome 7 are given negligible recurrence risk as all reported patients with UPD7 are sporadic [14, 50]. The monogenic pathogenic variants in *CDKN1C*, *IGF2*, *PLAG1* and the non-imprinted *HMGA2* gene carry high recurrence risk [50]. In our patient 2 WES reported frameshift variant in HMGA2 gene, (NM_001300918.1:c.310dup), p.(His104Profs*12), heterozygous, the variant is de novo and was classified as PM2 VUS.

Patients who carry copy number variants (CNVs) or single nucleotide polymorphisms (SNPs) in the region of imprinted genes can transmit them in an autosomal dominant pattern, depending on the imprinting condition of the affected gene. The clinical outcome depends on the sex of the parent carrying the affected allele. In our patient 2 the mutation in HMGA2 carries 50% recurrence risk in her offspring.

In PWs, patients with UPD, although the recurrence risk is low, if there is a parental Robertsonian translocation, the risk of UPD recurrence increases due to trisomy rescue [27]. For patients with a UPD of chromosome 14 or 15, chromosomal analysis of the parents should be performed. Prenatal testing for UPD is recommended in families with structural abnormalities in chromosomes carrying imprinted regions [51], we performed karyotype analysis in patient 3 and her parents for proper genetic counseling, the karyotype was normal in patient 3 and her parents, for patient 3 family the recurrence risk is low.

Methylation defects (epigenetic) are typically not inherited but occur sporadically. Recent studies have highlighted the secondary effects of certain CNVs near imprinting centers that can affect methylation.

Children with Beckwith-Wiedemann syndrome (BWS) have an increased risk of cancer. The highest cancer incidence in BWS children is with IC1-GOM (28.6%) and paternal uniparental disomy (UPDpat) (17.3%) [12]. Another study reported the highest cancer risk was associated with UPDpat, which exceeds the risk linked to IC2-LOM [52]. Additionally, duplication of the paternal 11p15.5 region carries a significant cancer risk [15]. Although our two BWS patients, who showed hypomethylation of *KCNQ1OT1* (KCNQ-CR), have a low cancer risk, regular follow-up is recommended.

There is a strong need to raise awareness among clinicians about the broad spectrum of clinical manifestations of imprinting disorders, MS-MLPA testing should be a routine part in diagnosing children with clinical manifestations of ImpDis.

Recent advances in the treatment of imprinting disorders have focused on novel approaches that specifically target the underlying epigenetic mechanisms. Examples of these emerging treatment methods include CRISPR-based Epigenetic Editing, which allows for precise correction of epigenetic marks, Small Molecule Epigenetic Modulators, which aim to restore normal gene expression, and Antisense Oligonucleotides (ASOs), which target and silence non-coding RNAs. Additionally, Artificial Intelligence (AI) and Big Data are being used to predict and personalize treatment strategies. These methods show great promise, but accurate diagnosis of the specific cause of the imprinting disorder remains crucial for effective treatment [3, 6, 52, 53].

## Conclusions

It is crucial to diagnose ImpDis accurately, as understanding the exact cause of ImpDis is important for genetic counseling and personalized medicine. There is a strong need to raise awareness among clinicians about the broad spectrum of clinical manifestations of imprinting disorders. methylation testing should be a routine part of diagnosing children with manifestations of ImpDis.

## Data Availability

No datasets were generated or analysed during the current study.

## Abbreviations

ACMG: American collage of Medical Genetics
BMI: Body mass index
BWS: Beckwith-Wiedemann syndrome
CMA: Chromosomal microarray
DMRs: Differentially methylated regions
GOM: Gain of methylation
ICRs: Imprinting control regions
ID: Intellectual disability
IGF1R: Insulin-like growth factor 1 receptor
IGN: Imprinted gene network
ImpDis: Imprinting disorders
IR: Insulin receptors
IQ: Intelligence quotient
IUGR: Intrauterine growth restriction
LOM: Loss of methylation
MLID: Multilocus imprinting disorder
MRI: Magnetic resonance image
MS-MLPA: Methylation-specific multiple ligation-dependent probe amplification
OFC: Occipitofrontal circumference
PWS: Prader-Willi syndrome
SRS: Silver-Russell syndrome
TNDM: Transient neonatal diabetes mellitus
UPD: Uniparental disomy
WES: Whole exome sequencing
